# Silver Nanowire-IZO-Conducting Polymer Hybrids for Flexible and Transparent Conductive Electrodes for Organic Light-Emitting Diodes

**DOI:** 10.1038/srep34150

**Published:** 2016-10-05

**Authors:** Ho Jun Yun, Se Jung Kim, Ju Hyun Hwang, Yong Sub Shim, Sun-Gyu Jung, Young Wook Park, Byeong-Kwon Ju

**Affiliations:** 1 Display and Nanosystem Laboratory, College of Engineering, Korea University, Seoul 02841, Republic of Korea; 2 The Institute of High Technology Materials and Devices, Korea University, Seoul 02841, Republic of Korea

## Abstract

Solution-processed silver nanowire (AgNW) has been considered as a promising material for next-generation flexible transparent conductive electrodes. However, despite the advantages of AgNWs, some of their intrinsic drawbacks, such as large surface roughness and poor interconnection between wires, limit their practical application in organic light-emitting diodes (OLEDs). Herein, we report a high-performance AgNW-based hybrid electrode composed of indium-doped zinc oxide (IZO) and poly (3,4-ethylenediowythiophene):poly(styrenesulfonate) [PEDOT:PSS]. The IZO layer protects the underlying AgNWs from oxidation and corrosion and tightly fuses the wires together and to the substrate. The PEDOT:PSS effectively reduces surface roughness and increases the hybrid films’ transmittance. The fabricated electrodes exhibited a low sheet resistance of 5.9 Ωsq^−1^ with high transmittance of 86% at 550 nm. The optical, electrical, and mechanical properties of the AgNW-based hybrid films were investigated in detail to determine the structure-property relations, and whether optical or electrical properties could be controlled with variation in each layer’s thickness to satisfy different requirements for different applications. Flexible OLEDs (f-OLEDs) were successfully fabricated on the hybrid electrodes to prove their applicability; their performance was even better than those on commercial indium doped tin oxide (ITO) electrodes.

Recently, research in highly flexible, transparent, and conductive electrodes has grown rapidly with the increasing industrial demands for their application in various optoelectronic devices and displays^1^,^2^,^3^. For these purposes, transparent conducting oxides (TCOs) have been widely investigated. Particularly, indium-tin-oxide (ITO) has been most widely used in displays because of its superior optical and electric properties compared to other TCOs^4^,^5^. However, aside from these advantages, ITO is not suitable for plastic-based flexible devices because of its brittleness and it tendency to crack it into pieces when subjected to repeated bending^6^. In addition, on temperature-sensitive plastic substrates, the performance of ITO is limited to a certain degree because ITO typically involves a high-temperature process to achieve its optimum optical and electric properties at the same time^7^. Much effort has gone into research to replace the ITO in transparent and flexible electrodes. Potential alternatives based on materials such as conductive polymers (e.g. poly(3,4-ethylenediowythiophene):poly(styrenesulfonate) [PEDOT:PSS]), 2D/3D carbon allotropes (e.g. carbon nanotubes and graphene)^8^,^9^,^10^, and metal nanowires^11^,^12^,^13^ have been investigated. Among these, silver nanowires (AgNWs) have attracted attention as a most promising candidate for the electrodes because they not only exhibit excellent optical and electric properties comparable to ITO but also have mechanical flexibility^14^,^15^,^16^.

However, several intrinsic drawbacks of AgNWs must be overcome to utilise them as electrodes for optoelectronic devices. For application in organic light-emitting diode (OLED) devices, it is required to decrease the surface roughness of AgNWs, which leads to nonuniformity in electric properties and possible electrical shorts at the ends of disconnected wires. The large open areas between AgNWs must also be filled to ensure sufficient contact to active areas for efficient carrier injection. In addition, the relatively lower than expected electrical conductivity of AgNWs, which arises from poor interconnections at wire junctions that necessarily employ an insulating capping layer (e.g. polyvinylpyrrolidone [PVP]), must be further improved^17^. To remove these defects, heat treatment at over 200 °C is required, which is not suitable for plastic substrates. Several alternative attempts have been made to reduce contact resistance and to produce large interconnected wires, such as light-induced junction welding or joule heating techniques. These methods efficiently reduce the junction resistance and improve non-uniformity in electric resistance by localised heating; however, the aforementioned surface roughness, which prevents sufficient contact with the other device layers, remains unsolved. To reduce the surface roughness of the films, several approaches, such as embedding AgNWs directly in polymer substrat or combining AgNW-based composite-type transparent conductive electrodes (TCEs) with PEDOT:PSS, metal oxides, graphene, or even metal oxide nanoparticles (NPs)^18^,^19^,^20^,^21^,^22^,^23^ have been reported. The former approach enables a highly smooth surface but requires costly processes, such as laser ablation, and expensive substrates to endure high temperatures and the etching processes. In addition, the embedding methods bury AgNWs inside the polymer matrix and only a small area is exposed at the surface, resulting in a limited conductive pathway, and consequently the charge injection into the active OLED layer is poor. Over-coating AgNWs with a conductive medium fills the gap between AgNWs, and some of methods were proven to tighten the wire junctions by welding, which results in improvements in carrier conduction. Among various over-coating materials, PEDOT:PSS is most commonly used because it has high transparency and a high work function, and it can smooth the surface morphology by penetrating through the AgNW gaps. However, PEDOT:PSS is proven to be detrimental to AgNWs, leading to poor stability of the composite films because of the water absorption and acid corrosion of AgNWs^24^. In our previous work, we successfully fabricated AgNW-based electrodes with indium zinc oxide (IZO), and demonstrated its suitability as a protective and conductive medium for AgNWs^25^.

Therefore, in this study, we developed new hybrid electrodes on plastic films by incorporating IZO between the AgNW and PEDOT:PSS layer. The IZO, unlike ITO, can be processed at a low temperature and works as a protective layer for AgNWs from the PEDOT:PSS and other oxygen-involving processes. In addition, IZO tightens the wire junctions and binds AgNWs to the substrate, which results in a significant reduction in contact resistance. The PEDOT:PSS for electrodes effectively reduces the surface roughness, and even increases the transmittance of hybrid films. The hybrid electrodes show improved current-stress stability and withstand the mechanical adhesion test without a loss in conductivity. A bending test of the hybrid TCEs confirmed that combining IZO does not deteriorate the bending performance, but retains its stability during and after bending, comparable to AgNWs by forming a buckled structure. A detailed investigation of the structure-property relations of the AgNW-IZO-PEDOT:PSS was conducted to find the optimum conditions. Herein, low sheet resistance and high transmittance spectra at certain wavelengths were realised with the most commonly used coating methods of spin coating and the sputtering for these materials; however, utilization of these materials in the roll to roll (R2R) process has been previously demonstrated by other studies^26^, indicating further improvements can be achieved through other potential methods. Furthermore, the hybrid electrode as an anode for flexible OLEDs (f-OLEDs) was demonstrated to show its applicability to real devices, and it performed better than reference devices with conventional ITO electrodes.

## Results and Discussion

**Figure 1:**
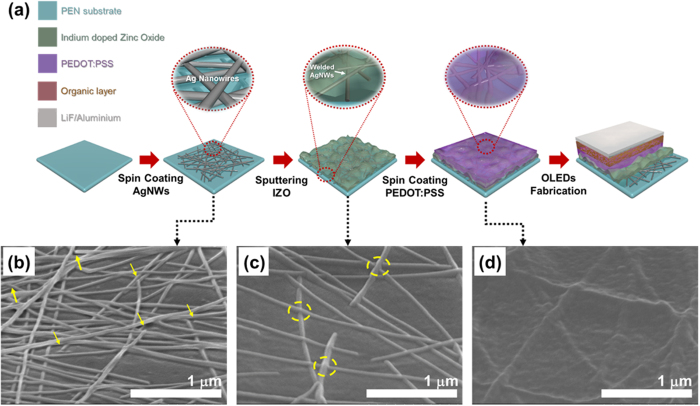
(**a**) Schematic diagrams of the fabrication process of the hybrid electrode and OLEDs. SEM images of AgNW hybrid electrodes on PEN: (**b**) 0.4 wt% pristine AgNWs, **(c**) AgNW-120-nm IZO hybrid electrode, (**d**) AgNW-IZO-70-nm PEDOT:PSS hybrid electrode. (The disconnected wires and welded wire junctions are identified by arrows and circles, respectively).

The fabrication procedure for the AgNW-IZO-PEDOT:PSS hybrid film is schematically illustrated in Fig. 1(a). The resulting three kinds of conductive films were investigated for quantitative analysis on the role of each of the materials, which were AgNW, AgNW-IZO, and AgNW-IZO-PEDOT:PSS. The morphologies of the hybrid films prepared with 0.4 wt% AgNW solution were characterised by scanning electron microscopy (SEM), as shown in Fig. 1(b–d). The SEM image demonstrates that randomly cross-linked AgNWs are loosely stacked, and steep spikes of wire ends are apparent. After deposition of the IZO layer, the entire AgNW network seems to be encapsulated in IZO. The sputtering of IZO on AgNWs inevitably involves high temperature (approximately 100 °C) heating, however, underlying AgNWs become highly thermostable due to thin layers of as-deposited IZO and commonly reported heat welding effect is not observed as shown in Supplementary Fig. S1. Below 200 °C range, sheet resistance decrease mainly due to thermal desorption of organic residues around AgNWs and their junctions including residues left from IPA evaporation^27^. Therefore, improvements on the sheet resistance (R_s_) of the AgNW networks is ascribed to both instantaneous evaporation of solvent and the cross junctions of the AgNWs which are welded tightly by IZO, bridging the charge transport across adjacent AgNWs. The additional PEDOT:PSS layer appears to homogenously infiltrate into the gaps and further improves the roughness of the AgNW-IZO films. In the fabrication process, the PEDOT:PSS, which by itself is a poor adhesive, requires other additives or surface treatments to promote adhesion for a sufficiently thick coating. The as-prepared AgNW and AgNW-IZO film was treated with an O_2_ plasma to improve the hydrophilic property during the process^28^. Moreover, the plasma treatment is proved to enhance the injection efficiency of holes for a high work function anode, which is beneficial for application in OLEDs^29^. However, AgNWs are known to be susceptible to oxidation and heat.

**Figure 2:**
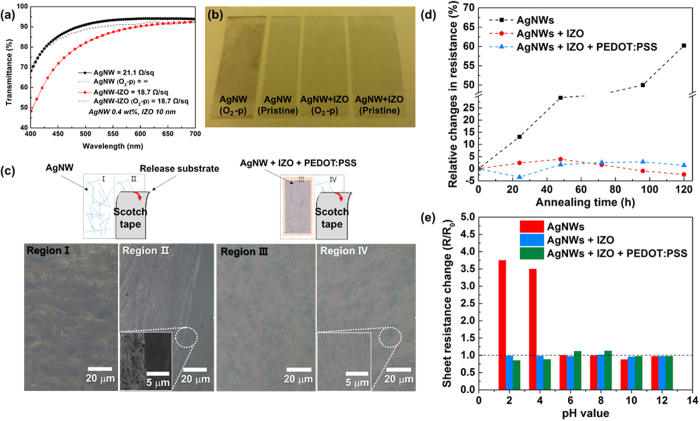
(**a**) Transmittance spectrum and (**b**) relative photographs of O_2_ plasma treated and non-treated pristine AgNW and AgNW-IZO hybrid films; O_2_-p denotes plasma treated samples. (**c**) Optical microscope (OM) images of the AgNWs and the hybrid films on the released substrate before and after the adhesion test using Scotch tape. (**d**) Resistance variation of AgNW based films when exposed to temperature of 85 °C and (**e**) when immersed in solution with different pH values.

To ensure that IZO can effectively encapsulate AgNWs from oxidation, and if possible, to determine the limit of the IZO thickness, the IZO thickness was controlled to 10 nm and as-prepared AgNW-IZO hybrids were treated with O_2_ plasma at 90 W power and 500 mTorr pressure for 20 min. After the treatment, as shown in Fig. 2(a), the corroded AgNW films’ sheet resistance increased from 21.1 Ωsq^−1^ to nearly insulator, and in addition to the increment in sheet resistance, approximately 5% transparency loss, when compared to unoxidized samples, was observed. In contrast, the AgNW-IZO film exhibited excellent optical performance in the visible spectrum without noticeable changes in sheet resistance. As shown in Fig. 2(b), the background is clearly visible for the transparent IZO coated electrode, whereas the pristine AgNW sample subjected to even 1 s of oxygen plasma is partly opaque because of the atmospheric corrosion of the silver by oxidation. These results show the efficacy of thin IZO in protecting the underlying AgNWs from oxidation and that the fabrication process is organised in a valid order. The strong attraction between the AgNWs and the coated IZO and PEDOT:PSS layers was confirmed via a Scotch tape test as schematically described in Fig. 2(c). The pristine AgNW film shows partly detached regions from the substrates, whereas most regions remain intact on the AgNW hybrid film. Furthermore, investigations on chemical stability of films by 5 days exposure to high temperature of 85 °C and 30 minutes immersion to solutions with various pH values were conducted. As shown in Fig. 2(d,e), the hybrid electrodes show superior chemical stability to that of pristine AgNWs since the variation of sheet resistance were much smaller for hybrid electrodes in both cases, and the morphology of hybrid films show no distinct changes while that of pristine AgNWs show defects induced by corrosion as shown in Supplementary Fig. S2.

The R_s_ and transmittance (T) of the AgNW hybrid films are closely related to the AgNW density and IZO thickness. To determine the optimum AgNW density for the AgNW film in the AgNW-IZO-PEDOT:PSS hybrid TCEs, first, we varied the AgNW density by preparing AgNW dispersion with four different weight percentages. The R_s_ and T values at 550 nm were used to calculate the figure of merit (FoM, *Φ*_TC_) as defined by the Haacke equation^30^:


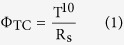


**Figure 3:**
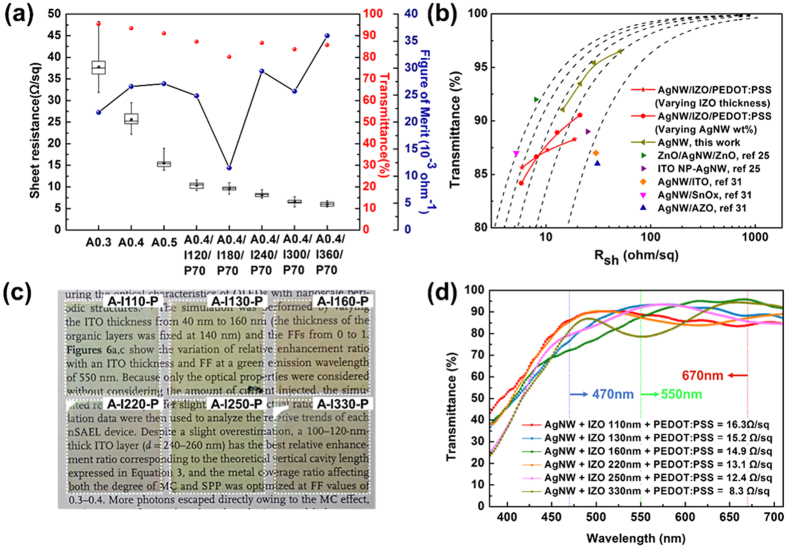
(**a**) FoM values for relative sheet resistance and transmittance values of the AgNW-based hybrid films (A : AgNW, I : IZO, P : PEDOT:PSS). (**b**) Transmittance (λ = 550 nm) as a function of the sheet resistance R_s_ of hybrid films (red symbols) with various AgNW densities and IZO thicknesses. A series of dotted lines represents the curves according to the Tinkham equation corresponding to σ_Op/σ_DC values of 100, 200, 300, 400, and 500. (**c**) Comparative photographs of the hybrid films with various IZO thicknesses (A: 0.3 wt% AgNW, I: IZO, P: 70 nm PEDOT:PSS). IZO thickness of 110 nm and 220 nm for blue, 130 nm and 250 nm for green, and 160 nm and 330 nm for red transmittance peaks. (**d**) Relative transmittance spectra and sheet resistance of each respective film.

Figure 3(a) depicts the R_s_ and calculated *Φ*_TC_ of AgNW films as a function of weight percentage as well as the *Φ*_TC_ of the hybrid electrodes according to the IZO thickness. The electrical properties of hybrid films were improved as AgNW density and IZO thickness was increased. The *Φ*_TC_ values increase with increasing AgNW weight percentage up to 0.5 wt%, which corresponds to increased surface coverage of 43%. The maximum *Φ*_TC_ value was 27.1 × 10^−3^ Ω^**−**1^ (T: 91.1%, R_s_: 14.5 Ωsq^−1^) when the weight percentage was 0.5 wt%. The performance of the AgNW films with weight percentages of 0.4 wt% and 0.5 wt% are almost identical in terms of FoM values, which implies that more values such as surface roughness and bending performance should be considered later in the study. In terms of hybrid films with 0.4 wt% AgNWs, the *Φ*_TC_- values vary significantly with IZO thickness, and accordingly, some values are much greater than for pristine AgNWs because the IZO transmittance shows peaks at certain wavelengths owing to the interference effect of incident light in the film^31^. Moreover, as shown in the graph with box-and-whisker plots (from the measurement of 16 different points), the homogeneity of the R_s_ was improved with the additional IZO and PEDOT:PSS layer. The interquartile range (IQR) values (defined as first quartile subtracted by third quartile of data) were reduced to 0.7 Ωsq^−1^ for the hybrid film with 360-nm IZO, while that of 0.4 wt% AgNW was 3 Ωsq^−1^. To optimise the thickness of IZO, the transmittance of IZO film was further investigated using finite domain-time domain (FDTD) analysis. The 2D colour map of IZO transmittance as a function of IZO thickness and wavelength is depicted in Supplementary Fig. S3. In the range of IZO thickness below 500 nm, IZOs of 120 nm, 240 nm, and 360 nm thickness showed high transmittance peaks at the 550-nm wavelength, which was the reason for the higher *Φ*_TC_ values in the hybrid electrodes. The transmittance spectra of each respective hybrid film with various AgNW concentrations, IZO thicknesses, and PEDOT:PSS thicknesses are summarised in Supplementary Fig. S4. In all hybrid films, the transmittance decreases with an increase in AgNW concentration, along with a rapid drop at short wavelengths due to absorption of AgNWs by surface plasmons^32^, although no serious decrease of transmittance is observed with a thin PEDOT:PSS layer. Interestingly, the average transmittance of AgNW-IZO increases with the introduction of PEDOT:PSS as shown in Supplementary Fig. S5. This can be ascribed to the improvement of the rough surface of the AgNW-IZO film^33^. Although the specular reflectance of a perfectly flat surface and the diffuse transmittance of a highly rough surface can cause a reduction in transmittance of the composite films, the slightly rough surface of the hybrid film (R_rms_ of 4.3 nm) could lead to the reduction of the reflection.

For a clear comparison of the R_s_ and T characteristics, plots of T versus R_s_ at λ = 550 nm are presented in Fig. 3(b). The optimal previously reported results for AgNW transparent conductors with various metal oxide layers are also shown. The relationship between T and R_s_ for a thin metallic film can be expressed by the Tinkham formula^34^:


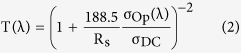


where 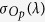 is the optical conductivity (at 550 nm) and 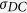 is the DC conductivity of the film^35^,^36^. The 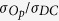 ratio is used as another FoM for an intuitive comparison of the R_s_-T plots. A series of dotted lines represents the curves fitted according to equation (2) when 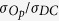 corresponds to 100, 200, 300, 400, and 500 as illustrated. A value of 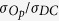 = 500 is observed for the conventional ITO film and the high-performing nano-structured thin film^37^,^38^. The results show that our AgNW-IZO-PEDOT:PSS hybrid electrode is comparable to the state-of-the art AgNW transparent electrodes^37^ and other AgNW-based hybrid electrodes^33^,^39^. The reduction in R_s_ (from 20 Ωsq^−1^ to 5.9 Ωsq^−1^) is more evident than the loss in transmittance (from approximately 88% to 86%) in the hybrid film with changes in IZO thickness, as intended. All the experimental values fell within the range of 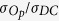 = 100–400. The value of 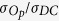 was higher than the minimum required value for the industry^40^.

In Fig. 3(c), photographs of AgNW-IZO-PEDOT:PSS hybrid electrodes of six different IZO thicknesses on polyethylene naphthalate (PEN) substrates are shown. While AgNW weight percentage and PEDOT:PSS thickness are fixed to 0.3 wt% and 70 nm, respectively, the IZO thickness is selected for the purpose of realizing high transmittance at 470-nm blue, 550-nm green, and 670-nm red, and each respective T spectrum and R_s_ is presented in Fig. 3(d). This result implies that with precise control of the layer thickness, more-conductive hybrid electrodes are possible with minimised loss in T at certain visible wavelengths, and this will be beneficial for the fabrication of OLED luminaire devices. The film appears somewhat colourful because of the sharp transmittance drop at short the wavelength region ascribed to the IZO layer; however, it is revealed that by controlling the zinc content in the IZO, the transmittance at short wavelengths can be improved^41^. An investigation into this mechanism in a subsequent study would further enhance the transmittance of the hybrid films.

**Figure 4:**
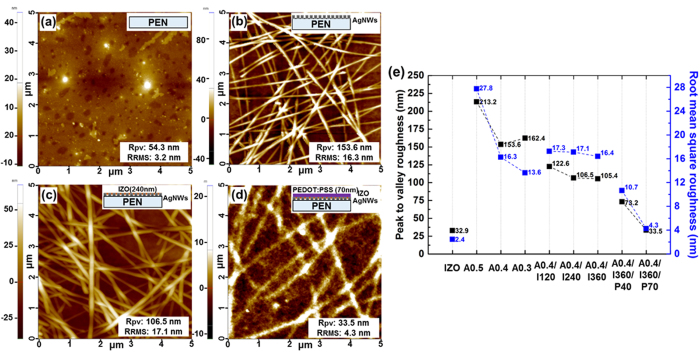
Comparison of morphologies by AFM analysis: (**a**) PEN substrate, (**b**) Ag nanowires on PEN, (**c**) IZO coated on the sample from (**b**,**d**) PEDOT:PSS coated on the sample from (**c,e**) a diagram comparing the roughness of various samples.

It is known that the surface morphology of the electrodes significantly affects the device performance because the interfacial contact with the subsequently coated organic layers on top of the electrode tends to degrade with increasing R_rms_^42^. Hence, the highly porous surface morphology of AgNW is not advantageous for thin-film organic devices. The morphologies of various samples were compared, including bare PEN substrate, AgNWs coated on PEN, AgNW-IZO composite film, and AgNW-IZO-PEDOT:PSS hybrid film, as shown in Fig. 4. As shown in Fig. 4(a–d), the deposition of the IZO layer is not particularly efficient in decreasing the R_rms_ of the AgNW film. However, it is revealed that R_pv_ of the surface was decreased, despite the small change in R_rms_. With the incorporation of the IZO film, disconnected ends of AgNWs are shown to be covered and smoothed, which will be effective in preventing vertical short-circuits in the fabricated thin-film devices. Intuitively, the surface states are expected to be further improved by a thicker coating of each material; however, the quantitative analysis is lacking on the thickness of IZO and PEDOT:PSS and the density of AgNWs. The R_pv_ and R_rms_ roughness changes of the four different types of films are summarised in Fig. 4(e): bare IZO, bare AgNW film, AgNW-IZO composite film, and AgNW-IZO-PEDOT:PSS hybrid film. Obviously, both the R_pv_ and R_rms_ of bare AgNW film tends to decrease when using smaller weight percentage AgNWs, and a steep decrease is observed between 0.5 wt% and 0.4 wt% AgNWs, whereas 0.4 wt% and 0.3 wt% shows little difference in R_pv_. Because each piece of AgNW has a diameter of approximately 30 nm, the results of 0.4 wt% AgNW having a R_pv_ of 153.6 nm is reasonable with the atomic force microscopy (AFM) image in Fig. 4(b), showing stacks of more than four wires. Hence, it can be said that using 0.4 wt% AgNWs, instead of 0.5 wt% AgNWs with a higher FoM is reasonable in terms of surface roughness. The relative surface roughness of AgNW-IZO composite with changes in IZO thickness shows that, in the ranges of our intended thickness (~360 nm), no dramatic reduction in surface roughness is caused by deposition of thicker IZO. Rather than IZO, the random network of AgNWs in the calculated AFM regions could be more responsible for the small changes in surface roughness. In order to clarify the effect of IZO on smoothing the surface, thicker IZO was deposited to the limit where clear reduction of surface roughness is observed. It can be expected from Supplementary Fig. S6 that the distinct effect in planarization can be observed only after sufficient deposition of the IZO over 650 nm. The surface morphology is further improved with the addition of the PEDOT:PSS layer, resulting in a R_pv_ of 33.5 nm and the R_rms_ of 4.3 nm, which is suitable for an OLED bottom electrode. In addition, these values are comparable to the values of sputtered IZO with a R_pv_ of 32.9 nm and even better than that of intrinsically rough commercial PEN substrate.

**Figure 5:**
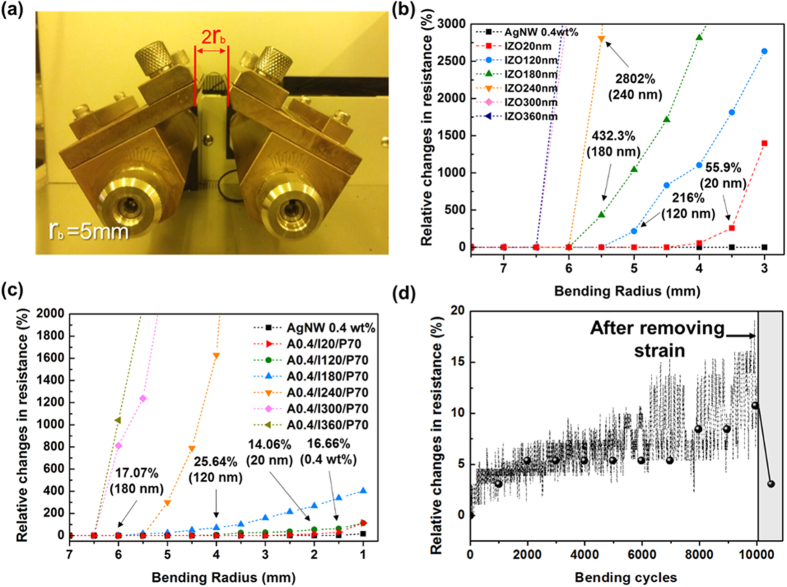
(**a**) Photographs of the bending tester and static outer bending test results for (**b**) bare IZO films and (**c**) AgNW-IZO-PEDOT:PSS hybrid films. (**d**) Cyclic outer bending test results at r_b_ = 5 mm for A0.4/I120/P70 film.

To test the mechanical durability of the films and the effects of increasing thickness of the IZO and PEDOT:PSS layer on bending performance, the hybrid films were subjected to bending at various bending radii (r_b_) whose curvatures were calculated as illustrated in Fig. 5(a). Figure 5(b,c) shows the change in resistance as a function of bending radius; the change in resistance is expressed as a percentage by (R − R_0_)/R_0_, where R is the resistance after bending and R_0_ is the initial resistance. All IZO films show little change in R_s_ above r_b_ = 7 mm. Note that the thickness of the hybrid film (including single-material film) is much thinner than that of the plastic substrate (~125 μm of PEN) in this study, so assuming that the film undergoes a similar degree of mechanical strain to that of the substrate is reasonable. The amount of strain applied to a film when bent with a r_b_ is estimated by^43^


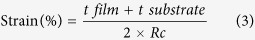


where t_film_ and t_plastic_ are the thicknesses of the hybrid film and the substrate, respectively. Although the degree of increase differs according to the thickness, IZO films with thicknesses ranging from 180 to 300 nm show a remarkable increase in resistance and become insulators near r_b_ = 6 mm, which corresponds to approximately 10% strain. The critical bending radius (r_b_^crit^), below which the change in R_s_ becomes larger than 10%, is marked to compare the bending stability. The r_b_^crit^ of 120-nm IZO is above 6 mm, whereas the r_b_^crit^ of hybrid films with the same thickness is 4 mm. According to the test results in Fig. 5(b), the r_b_^crit^ of bare IZO films show decreases with the introduction of AgNW and PEDOT:PSS. A cyclic bending test of the hybrid film with a 120-nm-thick IZO layer was performed at a fixed r_b_ of 5 mm for 10,000 cycles, which is known as the industrial standard to evaluate the bending stability of TCEs. In hybrid TCEs, an approximately 10% deviation in the sheet resistance was observed during the bending, where a 10% increase in the R_s_ i_s_ approximately 2–3 Ω/sq.

**Figure 6:**
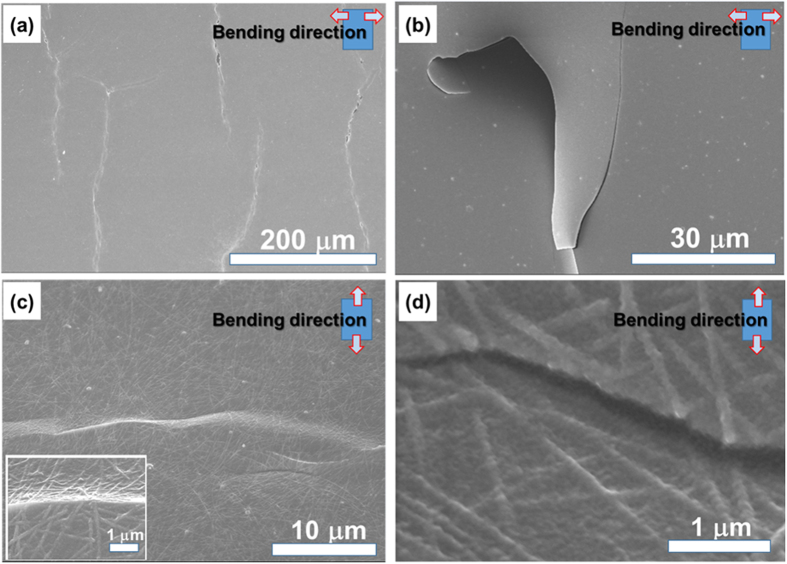
FE-SEM images showing the crack propagation patterns in (**a**) 120-nm-thick bare IZO, (**b**) IZO-PEDOT:PSS film, (**c**) AgNW-IZO-PEDOT:PSS hybrid film at r_b_ = 5 mm, and (**d**) AgNW-IZO-PEDOT:PSS films after 10,000 cycles at the same r_b_ as (**c**).

It is worth noting that the resistance of the hybrid film returns to almost its initial sheet resistance when released, whereas the IZO film shows no such behaviour, as shown in Fig. 5(d). SEM analysis was performed on the microstructure during and after bending to investigate this phenomenon. As shown in Fig. 6(a,b), the mode of cracking is similar in both bare IZO film and IZO-PEDOT:PSS film. The brittle cracks appear to propagate perpendicular to the bending directions, with PEDOT:PSS and IZO being detached and bent along the crack path. This observation explains the reason that bare IZO film becomes an insulator after the repeated bending process. However, the hybrid film with AgNW shows a small buckled structure because the tension induced by the bending propagates preferentially along the interfaces between IZO and AgNWs, as shown in Fig. 6(c)^44^. The PEDOT:PSS on the IZO also adheres tightly during the structural change and appears to maintain the conducting path of the hybrid film. This observation is consistent with other polymer-based electrodes forming buckles during bending^45^, and the formation of these nano-scale buckles appears to be responsible for the increase in resistance during the cyclic bending test as well as the distinct recovery in resistance after release. In addition, the strain and bending fatigue exceeding the limit of their structure seem to break through the wires rather than forming buckles, as shown in Fig. 6(d). On the basis of these test results, the bending stability of the hybrid TCEs is significantly better than that of the homogeneous IZO and even comparable to that of bare AgNW films.

**Figure 7:**
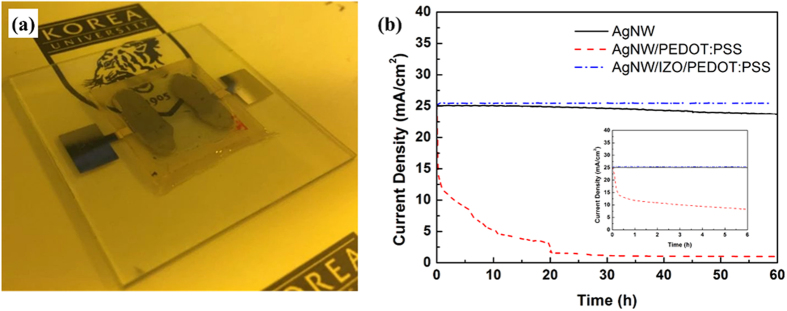
(**a**) Photo image of the fabricated sample for the current stressing test; (**b**) the measured current density changes over time for different conductive films.

Investigating the current stressing stability of the electrodes is also crucial to electrical device applications^46^. The injected current passing through the AgNW at some extent causes the electrical breakdown at wire junctions^47^. Furthermore, the hygroscopic and acidic properties of PEDOT:PSS give rise to the degradation of AgNW films, even at low current density, as reported by other studies^48^. Samples of AgNW, AgNW-PEDOT:PSS, and AgNW-IZO-PEDOT:PSS with an area of 1 cm^2^ were prepared as shown in Fig. 7(a). All AgNWs in hybrid films were pre-annealed at 100 °C for 10 min to reduce the effects of joule heating and remaining residues. A glass was used to encapsulate the hybrid electrodes to exclude the effects of oxygen and water in air, which can contribute to the degradation of the films. Different voltages were applied to the samples to apply an equal amount of stressing current (25 mA/cm^2^) for the test. Figure 7(b) shows the current density changes over time for the different films under fixed voltages, which is related to the conductivity changes during current stressing. For the bare AgNW film, the conductivity gradually decreases over time, which can be attributed to Ag migration at wire junctions or oxidations. The AgNW-PEDOT:PSS hybrid film shows a dramatic decrease in conductivity during current stressing, as expected. For the AgNW-IZO-PEDOT:PSS hybrid film, no signs of degradation are observed, even after 60 continuous hours of current stressing, demonstrating that IZO, as a protective layer, shows better operational stability.

**Figure 8:**
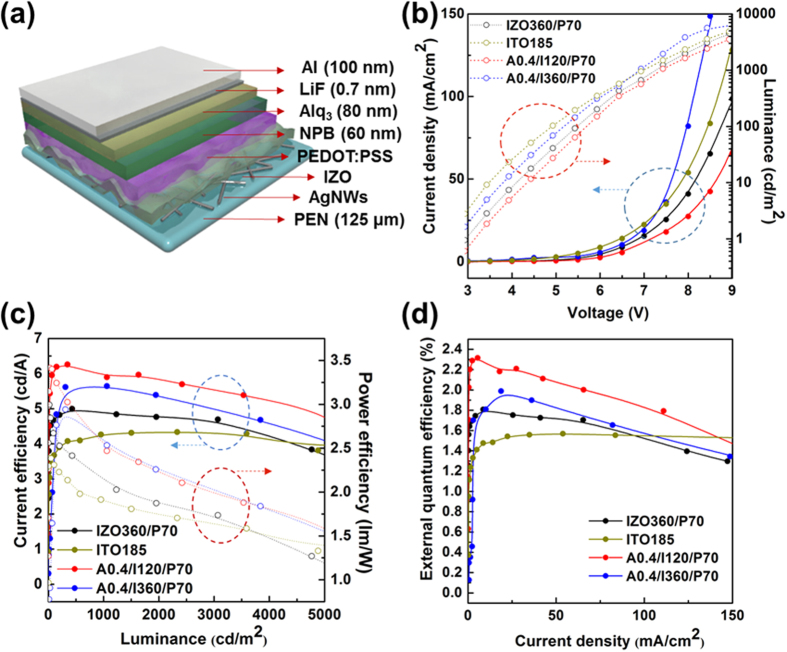
Device structure and EL characteristics of OLEDs on AgNW-IZO-PEDOT:PSS hybrid electrodes: (**a**) Schematic of the OLED structure, (**b**) current density and luminance versus voltage, (**c**) current and power efficiency versus luminance, (**d**) EQE versus current density.

In order to demonstrate the device compatibilities and evaluate the performance of the AgNW-IZO-PEDOT:PSS electrode, it was used in the fabrication of green fluorescent OLEDs with a device structure of TCE/N,N′-Di(1-naphthyl)-N,N′-diphenyl-(1,1′-biphenyl)-4,4′-diamine (NPB)/tris(8-hydroxyquinolinato)-aluminium (Alq_3_)/lithium fluoride (LiF)/aluminium (Al). An OLED device was also deposited on IZO-PEDOT:PSS and commercially obtained 185 nm thick ITO electrodes (10 Ωsq^−1^ with 87% transmittance at 550 nm) under the same conditions and used as a reference. Figure 8 shows the electroluminescence (EL) characteristics of the green fluorescent OLEDs based on different TCEs. Data for OLEDs based on pristine AgNWs are not shown because the device performance was poor and unstable because of high leakage currents and irregular charge recombination inside the emissive layer caused by large peak-to-valley roughness and the porous structure of the wires.

Unlike OLEDs devices based on AgNW electrodes exhibiting high leakage currents at small bias regions as reported by another study^49^, the OLEDs on the AgNW-IZO-PEDOT:PSS hybrid electrodes show low current leakage and enhanced stability. This might be due the IZO layer, preventing vertical shorts in the device because such leakage is also observed in the other study with an OLED device of same configuration, but without IZO^49^. The current density and luminance of the A0.4/I360/P70 device at bias >7 V becomes greater than the ITO reference device and each device’s current density and luminance were in the following order: A0.4/I360/P70 >ITO >I360/P70 >A0.4/I120/P70. In addition, the turn-on voltages of A0.4/I360/P70 (2.8 V) and A0.4/I120/P70 (3.2 V) were lower than that of I360/P70 (3.2 V) and comparable to that of the ITO reference. Supposedly, local current in the vicinity of the AgNWs reduced the turn-on voltage, and thicker IZO enlarged the active surface area of the sparsely dispersed AgNWs to adjacent layers^50^. The current efficiency of the devices on A0.4/I120/P70 (5.89 cd/A) and A0.4/I360/P70 (5.64 cd/A) were 38% and 32% higher, respectively, than the ITO (4.27 cd/A) reference at a brightness of 1000 cd/m^2^, indicating that the local current was advantageous in the hybrid electrodes. In addition, the PEDOT:PSS on IZO leads to a smaller current because of the poor conductivity, but leads to higher current efficiency, which suggests that the PEDOT:PSS layer can cover “bad” spots effectively as can be seen from the comparison of the OLEDs with AgNW-IZO and AgNW-IZO-PEDOT:PSS electrodes in Supplementary Fig. S7(a). The power efficiency of A0.4/I360/P70 (2.53 lm/W) was higher than that of A0.4/I120/P70 (2.46 lm/W), ascribed to the low operating voltage of the device on A0.4/I360/P70, but still higher than ITO (1.9 lm/W). The external quantum efficiency (EQE) of devices on A0.4/I120/P70 and A0.4/I360/P70 at 50 mA/cm^2^ were 2.08 and 1.80, whereas those of ITO and I360/P70 were 1.57 and 1.7, respectively. The transmittance of the fabricated hybrid electrodes at the Alq_3_’s peak EL spectrum of approximately 525 nm was 75.35% (A0.4/I120/P70) and 74.49% (A0.4/I360/P70), which was comparable to that of I360/P70, which was 81.64% with an approximately four-fold higher sheet resistance with the same IZO thicknesses. However, still higher transmittance of ITO (87.62%) suggests that the higher EQE of the hybrid electrode-based OLED devices can be due to the enhanced light extraction by light scattering of the AgNWs^51^. An efficiency roll-off was observed for all devices except the ITO reference. The comparison between OLED devices on IZO and IZO-PEDOT:PSS electrodes suggest that PEDOT:PSS could be the cause of the efficiency roll-off as presented in Supplementary Fig. S7(b). These overall results suggest that the AgNW-based electrodes could be applicable to OLED luminaires.

The comparable performance of the AgNW-IZO-PEDOT:PSS OLED and ITO/glass OLED with the same processing condition verifies the new hybrid film’s efficacy for use in flexible optoelectronic devices. It is expected that significantly improved performance can further be achieved with the improvement of AgNW morphologies and the optimization of the diode structure configuration.

In summary, we demonstrated highly conductive and flexible AgNW-based hybrid electrodes for use in OLEDs by combining AgNWs with sputtered IZO and PEDOT:PSS. Although AgNWs provide efficient conducting pathways, the deposited IZO on AgNWs serves effectively as a protective layer from oxidization and detrimental processes with no evident changes in their sheet resistance and transmittance. Tuning of the electrodes’ peak transmittance, which was not accompanied by typical trade-offs or significant changes in sheet resistance, were successfully demonstrated by varying each material’s conditions. The IZO and PEDOT:PSS worked as a glue to weld the wire junctions and adhere the AgNWs to the substrate, which prevented the AgNWs from possible separation from bending with significantly enhanced bending stability compared to commercial ITO, and they also exhibited excellent recovery of sheet resistance after physical stresses originating from their morphologies. Additionally, the PEDOT:PSS layer effectively reduced the surface roughness to an average roughness of 4.3 nm, even with an increase in transmittance and uniform electric conductivity. The hybrid films were shown to have enhanced stable performance under current stressing at a stressing current of 25 mA/cm^2^ for up to 60 h, whereas that of the reference reached half of its initial current density after only 1 h at the same conditions. The hybrid electrodes which were fabricated to have a peak transmittance at 550 nm had a transmittance of 86% with a sheet resistance of 5.9 Ωsq^−1^. The successful fabrication of f-OLEDs on the hybrid electrodes showed 32% EQE enhancements at 50 mA/cm^2^ compared to that of the ITO reference, which verified that the demonstrated electrodes in this work could be applied to OLED luminaires and offer high applicability for various flexible devices by replacing ITO.

## Methods

### AgNW/IZO/PEDOT:PSS Composite Electrode Fabrication

AgNWs were purchased from Aiden Co., Ltd. with an average diameter of 20–30 nm and length of 20–30 μm. A AgNW suspension was diluted in isopropyl alcohol to various concentrations (0.2–0.5 wt%) and was shaken with a vortex device for at least 1 min before using. PEDOT:PSS solution (CDT-H), mixed with a specially designed binder to increase resistance to dispersion of particles in water, while also making it soluble in an acidic liquid, was purchased from Ditto Co., Ltd. PEDOT:PSS was stirred for 20 min before using. The AgNW suspension with various concentrations was spin-coated onto PEN substrates or glass under a rotation speed of 1500 rpm, followed by a subsequent annealing process on a hot plate at 80 °C for 5 min to remove the solvent. IZO was then sputtered with a radio-frequency (RF) magnetron system under fixed conditions (power = 150 W, Ar flow rate = 3.5 sccm) for different times to obtain different thicknesses. The surface of the as-prepared AgNW/IZO films were treated with ultraviolet ozone (Ahtec LTS Co., Ltd.) and O_2_ plasma (Femto Science Co. Ltd) for a fixed time to obtain hydrophilic surfaces and cleaned surfaces for coating of PEDOT:PSS. PEDOT:PSS was then spin-coated at various rotation speeds, and the hybrid electrodes were then annealed at 100 °C to remove remaining solvent and enhance the π-π stacking of the conjugated polymer chains^52^. No further post-treatment was required.

### OLEDs Fabrication

The fabrication of bottom-anode hybrid electrodes followed the same procedures as described in the AgNW/IZO/PEDOT:PSS hybrid electrodes fabrication section. For the preparation of reference devices, commercial ITO (Samsung Corning Co., Ltd.) was purchased and ultra-sonicated to obtain clean surfaces. All electrodes were loaded in the same chamber. Active areas were defined by a shadow mask and organic materials were deposited under vacuum of 10^−6^ Torr, with thicknesses as the OLED device structure, followed by metal deposition using a thermal evaporator. NPB was used as the hole-transport layer, Alq_3_ was used as the emitting layer, LiF was used as the electron-injection layer, and Al was used as the cathode.

### Optical, Electrical, and Mechanical Characterizations

Transmittance, absorption, and reflectance were measured by employing an ultraviolet (UV)-visible spectroscope (Cary 5000, Varian). The sheet resistance measurement was carried out by a standard four-point probe system. The SEM images were obtained using an S-4800 (Hitachi Co., Ltd.). The electrical stressing test was conducted at a constant voltage using a high-voltage source measurement unit (Model 237, Keithley Instruments, Inc.). The EL characteristics of the fabricated OLEDs were measured using a high-voltage source measurement unit (Model 237, Keithley Instruments, Inc.) and a spectroradiometer (PR-670 SpectraScan, Photo Research, Inc.) Surface topology images of the hybrid electrodes were taken using an AFM (XE-100, Park Systems Inc.). A cyclic bending test was performed using a bending tester (z-tec, Inc.) with a digital multimeter to indicate the real-time line resistance.

## Additional Information

**How to cite this article**: Yun, H. J. *et al*. Silver Nanowire-IZO-Conducting polymer Hybrids for Flexible and Transparent Conductive Electrodes for Organic Light-Emitting Diodes. *Sci. Rep.* **6**, 34150; doi: 10.1038/srep34150 (2016).

## Supplementary Material

Supplementary Information
