# Supplementary material for: Silver Nanowire-IZO-Conducting Polymer Hybrids for Flexible and Transparent Conductive Electrodes for Organic Light-Emitting Diodes
Source: Sci Rep. 2016 Oct 5;6:34150. doi: 10.1038/srep34150 (PMC5050450; doi:10.1038/srep34150)
Supplement: Supplementary Information [file srep34150-s1.pdf]

Silver Nanowire-IZO-Conducting Polymer Hybrids for Flexible and Transparent Conductive Electrodes for Organic Light-Emitting Diodes

Ho Jun Yun[1], Yong Sub Shim[1], Sun-Gyu Jung[1], Young Wook Park[2,*], and Byeong Kwon Ju[1,]**

[1]Display and Nanosystem Laboratory, College of Engineering, Korea University, Seoul 02841, Republic of Korea,

[2]The Institute of High Technology, Materials and Devices, Korea University, Seoul 02841, Republic of Korea.

*zerook@korea.ac.kr, +82-2-3290-3665

**bkju@korea.ac.kr, +82-2-3290-3671

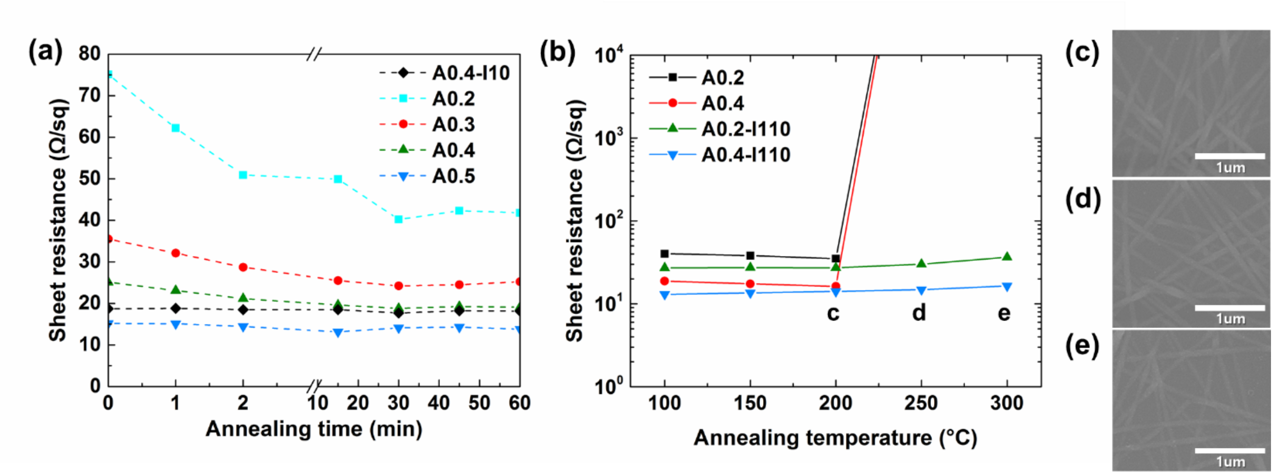

Supplementary Figure S1. (a) Variation of the sheet resistance of the AgNW based hybrid electrodes (A : AgNW, I : IZO) during a thermal annealing at 100 ℃ in air and (b) variation of the sheet resistance of the AgNW based hybrid electrodes with different annealing temperatures in air. SEM images show the morphology changes of the network of A0.4-I110 at different stages indicated in the graph by letters c, d and e: (c) 200 ℃ (d) 250 ℃ (e) 300 ℃. No evident differences are observed.

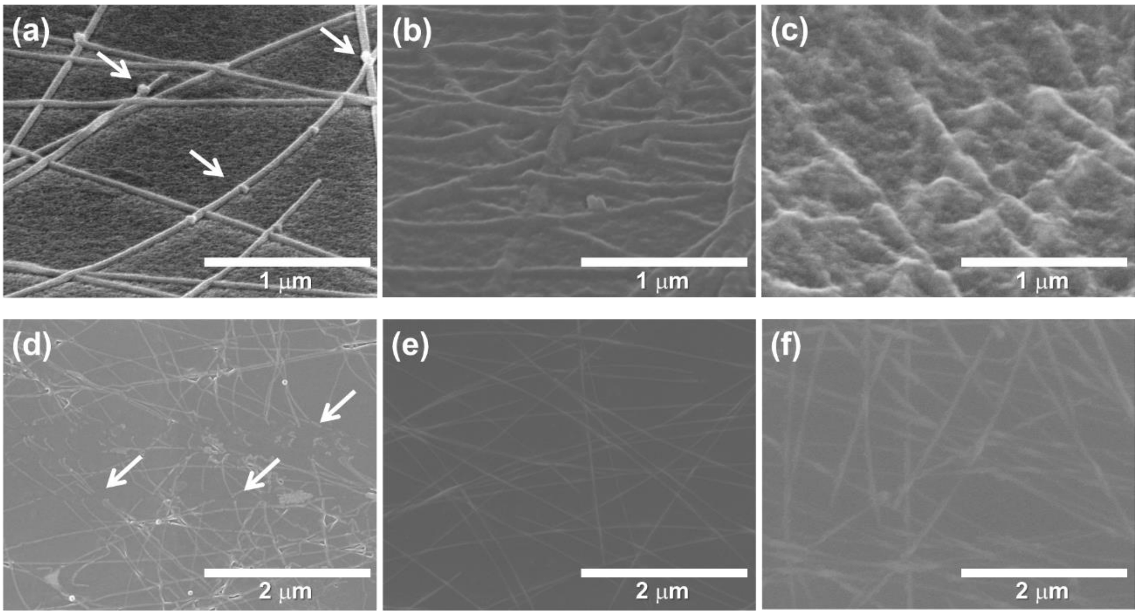

Supplementary Figure S2. SEM images presenting the surface morphology of (a, d) 0.4 wt% pristine AgNWs, (b, e) AgNW-110-nm IZO, and (c, f) AgNW-IZO-70-nm PEDOT:PSS hybrid electrodes after exposure for 5days to (upper row) high temperature (85 ℃) and (bottom row) after immersion for 30minutes to pH 2 solution. White arrows indicate defects induced by corrosion of AgNWs

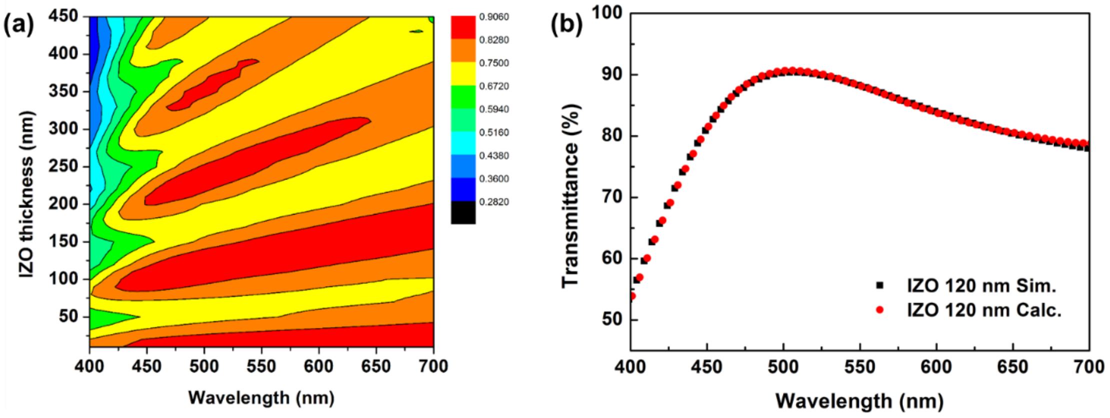

Supplementary Figure S3. (a) 2D colour contour map of simulated transmittance spectra of IZO as a function of IZO thickness and wavelength, and (b) transmittance spectra of simulated and experimental 120-nm-thick IZO film.

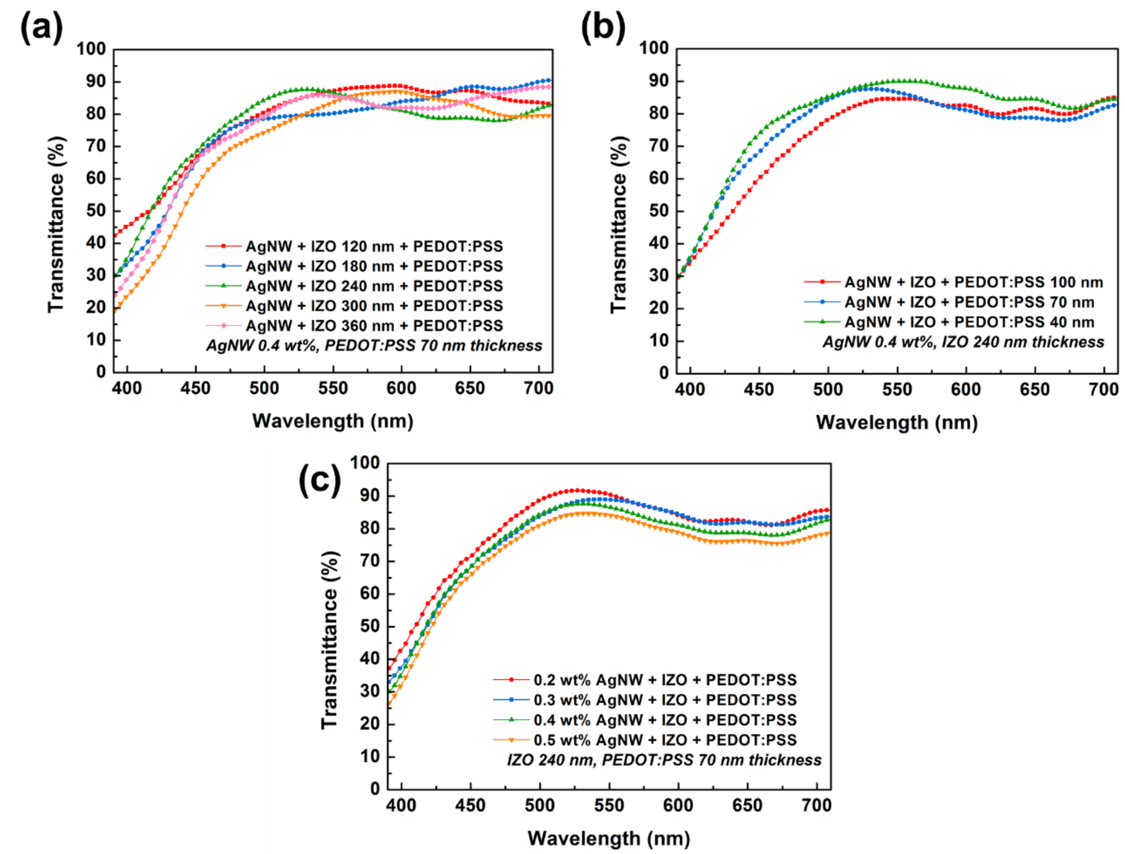

Supplementary Figure S4. Transmittance spectra of the AgNW-IZO-PEDOT:PSS hybrid films with various values of (a) IZO thickness , (b) PEDOT:PSS thickness, and (c) AgNW density.

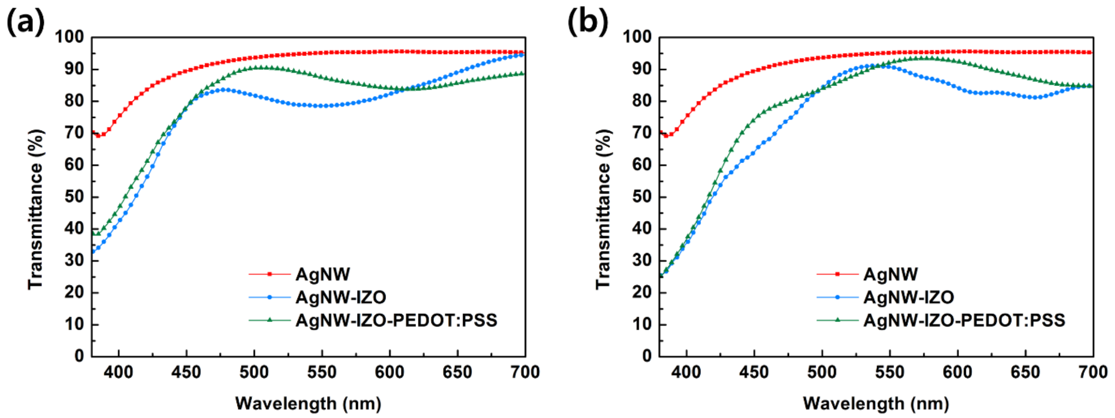

Supplementary Figure S5. Transmittance spectra of various AgNW base films: 0.3 wt% AgNW, AgNW coated with (a) 220-nm- and (b) 250-nm-thick IZO layers and AgNW-IZO films coated with 70-nm-thick PEDOT:PSS.

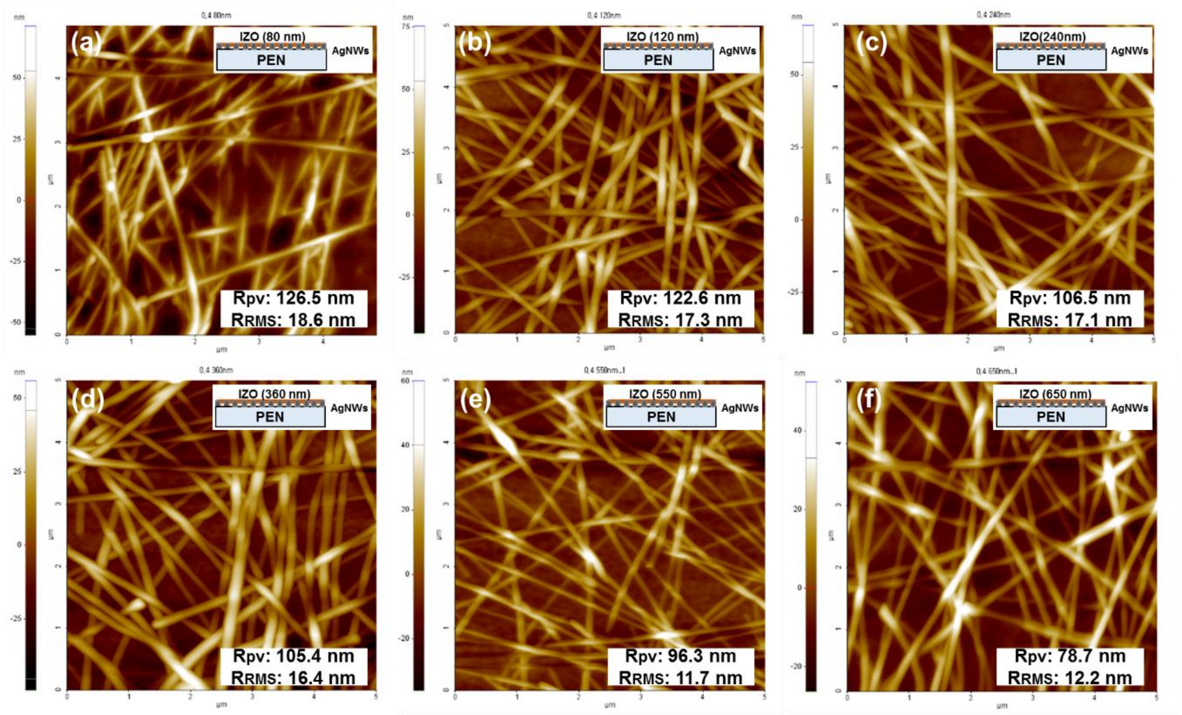

Supplementary Figure S6. Comparison of morphologies by AFM analysis: AgNW-IZO hybrid films on PEN with (a) 80-nm-, (b) 120-nm-, (c) 240-nm-, (d) 360-nm-, (e) 550-nm-, and (f) 650-nm-thick IZO layers.

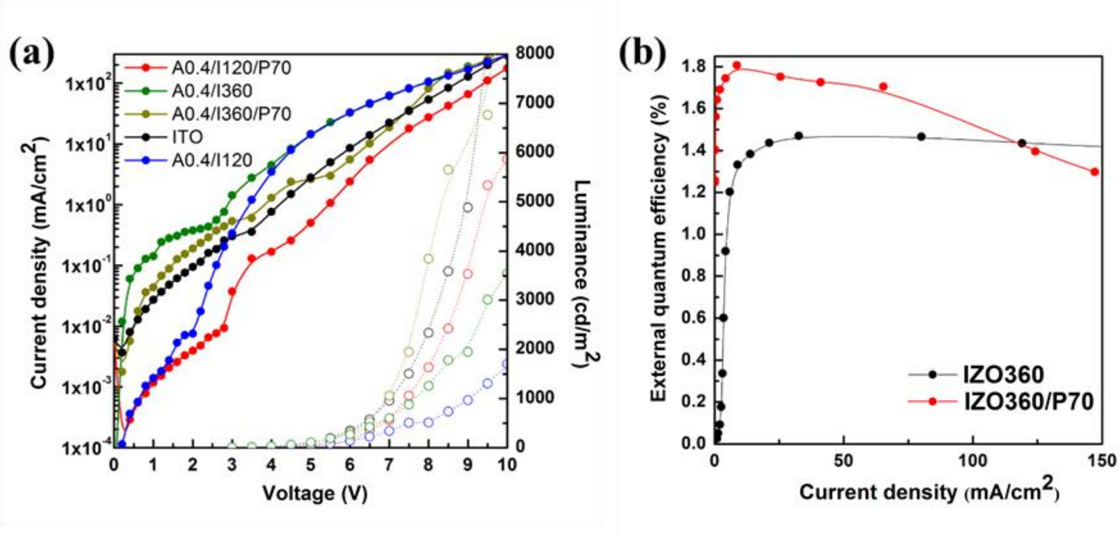

Supplementary Figure S7. (a) J-V-L characteristics and comparison of OLEDs on AgNW-IZO and AgNW-IZO-PEDOT:PSS electrodes. (b) J-EQE characteristics for OLEDs with bare IZO and IZO-PEDOT:PSS electrodes.
